# Exosomes: Small EVs with Large Immunomodulatory Effect in Glioblastoma

**DOI:** 10.3390/ijms22073600

**Published:** 2021-03-30

**Authors:** Laura Benecke, Mali Coray, Sandra Umbricht, Dapi Chiang, Fabrício Figueiró, Laurent Muller

**Affiliations:** 1 Department of Biomedicine, University of Basel, 4051 Basel, Switzerland; laura.benecke@usb.ch (L.B.); m.coray@unibas.ch (M.C.); dapi_chiang@biovesicle.com (D.C.); 2 Department of Otolaryngology and Head & Neck Surgery, University Hospital Basel, 4051 Basel, Switzerland; 3 Faculty of Medicine, University of Basel, 4051 Basel, Switzerland; sandra.umbricht@stud.unibas.ch; 4 Departamento de Bioquímica, Instituto de Ciências Básicas da Saúde, Universidade Federal do Rio Grande do Sul, Porto Alegre, Rio Grande do Sul 90035-003, Brazil; fabricio.figueiro@ufrgs.br

**Keywords:** extracellular vesicles, exosomes, biomarker, liquid biopsy, TEX, cancer, tumor immunology

## Abstract

Glioblastomas are among the most aggressive tumors, and with low survival rates. They are characterized by the ability to create a highly immunosuppressive tumor microenvironment. Exosomes, small extracellular vesicles (EVs), mediate intercellular communication in the tumor microenvironment by transporting various biomolecules (RNA, DNA, proteins, and lipids), therefore playing a prominent role in tumor proliferation, differentiation, metastasis, and resistance to chemotherapy or radiation. Exosomes are found in all body fluids and can cross the blood–brain barrier due to their nanoscale size. Recent studies have highlighted the multiple influences of tumor-derived exosomes on immune cells. Owing to their structural and functional properties, exosomes can be an important instrument for gaining a better molecular understanding of tumors. Furthermore, they qualify not only as diagnostic and prognostic markers, but also as tools in therapies specifically targeting aggressive tumor cells, like glioblastomas.

## 1. Background

Glioblastoma (GBM), a primary brain tumor in adults, is among the most aggressive tumors we know. Worldwide, 100,000 people are diagnosed with glioma each year [1] and most suffer from the highly aggressive form, glioblastoma (grade IV). In fact, it accounts for 52% of all primary brain tumors [1]. Despite enormous advances in the involved oncological disciplines, the prognosis of affected patients remains poor [2]. Patients still suffer from an aggressive course, and survival rates have improved only marginally over the last three decades [3,4]. Thus, the median overall survival from diagnosis to death is approximately 15 months [5]. The reasons for this aggressive behavior are the subject of intensive current research efforts.

One of many difficulties with this tumor entity is that the tumor is genetically and epigenetically very heterogeneous. Deep sequencing of the genome and transcriptome, as well as analyses of the epigenome, have shown a wide range of genetic and epigenetic variations within the same glioblastoma, so that the development of therapies to eliminate all tumor cells is challenging [6,7]. This genetic and phenotypic heterogeneity was also taken into account by the WHO classification of 2016 [8], which already incorporates certain mutations.

## 2. Glioblastoma and its Immunosuppressive TME

Glioblastomas establish a highly immunosuppressive tumor microenvironment (TME) [9], communicating with normal brain cells to create a microenvironment that supports tumor progression. This affects practically every cell in the TME.

Gliomas, and especially GBMs, take advantage of various communication pathways, these include growth factors, cytokines, chemokines, tunneling nanotubes, and microtubes, as well as extracellular vesicles [5,10], to name just a few.

This characteristic enables them to stimulate angiogenesis [11] or to use existing vessels for themselves [12]. They alter astrocytes in such a way that they contribute to tumor support [13] and even alter the extracellular matrix (ECM) [14] to facilitate invasion. 

The immune tumor micromilieu plays a pivotal role in tumor development and progression. We already know this from many other tumor entities [15] but it is particularly noteworthy in this tumor, for several reasons. For a long time, the brain has been considered to be immune privileged [16]. However, this concept has been challenged by some studies showing that there are interactions between both the central and peripheral immune systems [17]. For example, it has been shown that both antigens and T cells reach cervical lymph nodes through CSF-filled channels [18]. Second, up to one-third of the tumor mass is composed of microglia/macrophages [19], so it is worth taking a closer look at their role in tumor progression. Contrary to the first assumption, a high number of immune cells within the tumor microenvironment do not correlate with better survival. Inversely, a high proportion of immune cells are associated with tumor progression and reduced survival [20,21,22]. Immune cells, whose proper role should be to defend against tumors, are hijacked by the tumor to promote its progression. Glioblastomas are able to recruit immune cells from the blood, with monocytes making up the largest proportion. This cell type is characterized by high plasticity [23]. They are classified into M1 (pro-inflammatory functions) and M2 (anti-inflammatory functions) [24]. A growing number of studies have shown that naive macrophages, under the influence of the tumor microenvironment, become tumor-associated macrophages (TAMs), which both promote tumor growth and inhibit other immune cells [25,26]. Yet, the underlying factors of this transformation are not sufficiently understood. Just last year, Azambuja et al. [27] authored a publication showing that exosomes of glioblastomas seem to play an active role in this process.

## 3. Exosomes: The Smallest Group of EVs 

**Figure 1:**
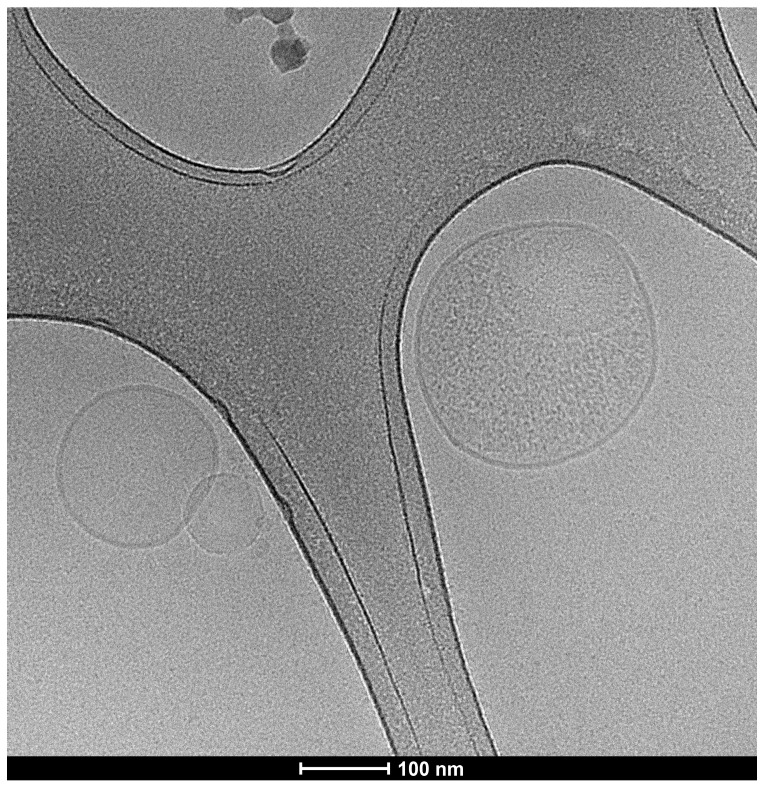
Representative image of freshly shock frozen samples in electron microscopy (cryo-electron microscopy) displaying the typical vesicular appearance with double lipid layer and size range of exosomes with 20,000× magnification.

Based on their size, biochemical properties, and origin, extracellular vesicles (EVs) are classified into three subgroups. These include apoptotic bodies, microvesicles, and exosomes [28]. The latter are the smallest group of EVs, comparable to viruses regarding their size (30–150 nm) (Figure 1) [29]. Exosomes are released by practically all cells and found in nearly all body fluids [30]. Exosomes differ from other EVs by their biogenesis through the endosomal compartment, thus carrying, for example, tsg101 as a typical marker [31]. They carry a lot of the molecular characteristics of the parental cell and due to their small size can reach nearly every part of the body. To illustrate this a little, we can compare exosomes to hemerodromes in ancient Greece. They delivered messages over long distances. The most famous one might be Pheidippides who covered around 240 km in two days to ask for military support. After this huge run, he is said to have collapsed and died [32]. Exosomes are also mostly used up after transmitting their message [31]. In order to keep the communication system running and to have a constant influence on the organism, and in particular on the immune system, tumor cells constantly produce large amounts of exosomes, called tumor-derived exosomes (TEX). They acquire lipids, proteins, and nucleic acids during their formation in the parental cell, which in some respects makes them a kind of miniature version of the mother cell [33]. This property in turn can be of great interest in order to characterize the properties of the tumor. For example, Thakur et al. demonstrated that exosomal DNA (exoDNA) represents the entire genome and reflects the mutational status of the parental tumor cells [34]. 

According to well-established scientific opinion and our own findings, we understand that exosomes are able to interact with immune cells in a way that aids in tumor immune escape. For example, in a paper published in 2017, we showed that TEX affects the suppressive activities of regulatory T cells (Tregs) by utilizing receptors on the surface of recipient cells [35]. In another study, we investigated how TEX are not internalized by T cells, but the signals they carry are delivered to cell surface receptors and thereby modulate the gene expression and functions of human T lymphocytes. Tregs were shown to be more sensitive to TEX-mediated effects than other T cell subsets [36].

## 4. Exosomes in the TME of Glioblastoma: Potent Modulators of the Immune Response

A typical feature of tumor progression is the inflammatory response in the TME with an accumulation of macrophages [15]. Depending on their activation status, they exert an anti-tumor or a pro-tumor effect. As mentioned above, pro-tumor M2 macrophages seem to predominate in the TME [37]. This knowledge, however, can only help us in the development of therapeutic approaches if we understand what causes this shift to the immunosuppressive form of macrophages. Gabrusiewicz et al. [38] showed an interesting effect of glioblastoma stem cell-derived exosomes. After uptake into macrophages, a reorganization of the actin cytoskeleton occurred, which resulted in a shift towards the immunosuppressive M2 phenotype, including expression of PD-L1. The authors concluded that glioblastoma stem cell-derived exosomes qualify as potent modulators of the immunosuppressive tumor microenvironment. 

This assumption was also supported by a recent study on the molecular and immunosuppressive properties of glioblastoma-derived exosomes (GDEs) [27]. For this purpose, exosomes from three human GBM cell lines were investigated. All exosomes obtained carried known immunosuppressive markers including CD39, CD73, FasL, CTLA-4, and TRAIL. Coincubation experiments with NK cells, CD4+ T cells, and CD 8+ cells revealed downregulation of activation status, reduced cytokine production, and enhanced apoptosis of CD8+ T cells. Human macrophages changed their phenotype and expression pattern after co-incubation with GDEs toward M2 macrophages with typical markers, including CD206, IL-10, LAP, and arginase-1. Other upregulated markers in this population were CD39, PD-1, and EGFR. The control group showed no change in expression. These observations were confirmed at the pathway level. To further substantiate the in vitro observations, GDEs were injected into normal mice and the concentration of CD8+ T cells and M1-like macrophages were measured in the spleen. Both showed a significant reduction, accompanied by an increase in M2-like macrophages. Juliana Azambuja and colleague [9] thus impressively demonstrated how exosomes from glioblastomas exert a suppressive effect on different types of immune cells.

This effect does not necessarily have to be exerted in a direct way. Rather, it is a kind of snowball effect, in which exosomes seem to be a driving force. This is also illustrated by data from the Pittsburg group around Theresa L. Whiteside [27], who showed in 2020 that exosomes from glioblastoma triggered a conversion of macrophages to tumor-associated macrophages (TAMs). TAM-derived exosomes, second-row exosomes so to speak, appear to play a significant role in tumor progression. Among the upregulated proteins of TAMs-derived exosomes, was arginase-1. Targeted arginase inhibition suppressed GBM proliferation mediated by TAM-derived exosomes. The authors concluded that blockade of arginase could be a potential therapeutic approach. Though, at the same time, they acknowledged that TAM-derived exosomes also carry markers that influence glioma cell migration, resistance, invasion, and other biological functions [27]. Yet, it is certainly an interesting player in the network of exosome-triggered tumor progression. Not least because disorders in L-arginine metabolism and their effect on both carcinogenesis and the activity of the antitumor immune system have recently become a focus of interest [39]. For example, Czystowska-Kuzmicz et al. [40] demonstrated that arginase-1 + exosomes from an ovarian cancer cell line reduced the proliferation of murine T cells and reduced the expression levels of CD3ζ and CD3ε chains. Therefore, it has already been suggested to investigate arginase-1 as a potential target in tumor therapy [39,41]. This is in contrast to a study by Bian et al., which showed that tumor-induced myeloid-derived suppressor cells (MDSC) do not generally express arginase-1, nor is this required for MDSC-mediated inhibition of T cells [42]. 

However, perhaps these results are only seemingly controversial. Thus, it is becoming increasingly apparent that the immunomodulatory effects often take complex detours and have limited reproducibility in in vitro models. Moreover, Domenis et al. [43] could show that the suppressive effect on T cells by GDEs is indirect via influencing monocyte maturation rather than directly interacting with T cells. Therefore, we still consider the role of arginase-1 positive exosomes to be an interesting approach. Indeed, numerous immunomodulatory molecules are found in exosomes isolated from glioblastoma patients. These include antigen-presenting molecules, tumor antigens, immune intracellular adhesion molecules, and TGF-ß [44]. These effects are not concentrated on only one group of immune cells, but affect all groups of immune cells [45,46]. In addition to macrophages, NK cells, and T cells, B cells also appear to be affected in their function [47]. 

## 5. B Cells in the TME of Glioblastoma: A Potentially Underestimated Immunocyte

To our knowledge, little is known about the role of B cells in gliomas and especially about the effect of exosomes on B cells in the tumor microenvironment. Colleagues from Ulm, Germany, recently studied the effect of circulating exosomes on B cells in HNSCC [48]. For this, they obtained B cells, as well as exosomes, from the peripheral blood samples of healthy patients, as well as those with head and neck squamous cell carcinoma (HNSCC). Like we also observed [49,50] the protein concentration of circulating exosomes was higher in the diseased patients than in the healthy patients. They concluded that plasma-derived exosomes show inhibitory effects on the function of healthy B cells. Notably, there was little difference in the inhibitory effects by exosomes between the two groups, assuming a physiological B-cell inhibitory role of circulating exosomes [48]. It is likely that the situation is similar in other tumor entities. Among the few other studies on this are those by Catalina Lee-Chang, published in December 2019 [47], where she and her colleges investigated B cell-mediated immunosuppression in glioblastoma. Forty percent of patients showed B cell tumor infiltration. This supports other studies, which have also previously shown that B cells are present in the tumor microenvironment [51]. Nevertheless, it remains to be clarified whether, and to what extent, they contribute to tumor progression.

To address this question, Lee-Chang et al. [47] studied human and mouse GBM-associated regulatory B cells (Bregs) with respect to their immunosuppressive potency on activated CD8+ T cells. Local application of B cell-depleting immunotherapy with CD20 antibodies resulted in highly significantly improved overall survival in animals, indicating a so far underestimated role of Bregs in tumor progression. Further investigation revealed an interesting mechanism. Thus, myeloid-derived suppressor cells (MDSCs) in the TME of glioblastomas appear to give membrane-bound PD-L1 via microvesicles to naive B cells, thus changing them toward the regulatory subtype. Upon uptake, these Bregs were able to inhibit CD8+ T cell activation, and thus contribute to tumor immune escape. CD163-bearing MVs from myeloid cells were also observed to be preferentially taken up by B cells, as well as Foxp3+ Tregs, a finding that implies that transfer of PD-L1-bearing MVs may be a universal process of intercellular communication between regulatory cells [47]. 

The contribution of B cells to tumor progression through tumor immune escape has long received little attention. This is due, not least, to the fact that B cells were for a long time regarded mainly as major effector cells of the humoral defense [52]. Not until recently have the antibody-independent functions of B cells received more attention. In a study published in 2016, we addressed the question of the extent to which B cells contribute to immune regulation. One of the difficulties of this topic is that the group of regulatory B cells is very heterogeneous and so far not a conclusively categorized subtype [53]. For quite some time now, one has begun to abandon the rigid classification into different groups of lymphocytes as the definitive form of differentiation. It rather seems that the different classes of lymphocytes are adaptations to the environment with high plasticity [35,36,53,54,55].

## 6. Adenosine (ADO) in the TME: Potent Mediator of Immunosuppression

ATP is considered the energy currency of the body and is found everywhere in the organism. Particularly high concentrations are found during increased cell turnover [56,57]. Under physiological conditions, the extracellular concentration is around 30–200 nM but may increase a hundredfold in the context of hypoxia, inflammatory reactions, or in the tumor microenvironment [56]. While extracellular ATP promotes inflammation and is able to recruit immune cells, ADO has an inhibitory effect on the immune system [58]. The purine nucleoside is involved in angiogenesis and suppression of the antitumor functions of effector T cells, while enhancing the functions of suppressor cells (Treg and MDSC) [59,60], resulting in tumor progression [61,62]. As ADO is abundant in the TME, due to cell death, it is thought to be one of the most potent mediators in the TME [63]. 

It was already observed that TEX have a significant effect on T cells by modulating them differentially [36,64,65,66]. For instance, TEX were previously reported to inhibit the functions of human activated CD8+ T lymphocytes by inducing their apoptosis via the Fas/FasL pathway [66]. A proprietary study could provide evidence for differential exosome-mediated changes in gene expression levels in resting vs. activated T cells [36]. Nevertheless, the impact on B cells is less well known, and deserves further attention, as B cells are, amongst others, central effector cells of the adaptive immune response [64,67,68]. It is well known that B cells utilize the adenosine (ADO) pathway [69]. As such, they express both CD39 and CD73; ectonucleotidases that can degrade extracellular ATP to ADO in two sub-steps. 

Current in-house studies, among others, shed light on the effect of tumor-derived exosomes (TEX) on the recently described CD39high B cell subtype in dependence on ATP concentration. The regulatory B cell subset is characterized by carrying high expression levels of the enzyme CD39 on their cell surface (CD39high B cells) [68,70,71]. This B cell subtype is thought to have immunosuppressive properties through secretion of anti-inflammatory cytokines such as IL-10, as well as inhibition of T effector cell in proliferation and activation. The data is currently under submission, but is mentioned here for completeness. 

Interestingly, CD39high B cell subset population increased upon activation of B cells with IL-4 and CD40L. Upon addition of TEX, the percentage of CD39+, as well as CD39high B cells, in the total B cell population decreased. However, CD 39high B cells expressed more CD39 per B cell after co-incubation with TEX. Furthermore, TEX was also shown to affect the expression of apoptosis-associated proteins in activated B cells. 

The best studied enzymes required to degrade extracellular ATP to ADO are CD39 and CD73. It has repeatedly been shown that CD39 and CD73 may be overexpressed in cancer cells, but also in various subsets of immune cells and stromal cells. The resulting increase in adenosine concentration in the TME leads, among other things, to an impairment of antitumor immunity [72]. CD39 has been considered the most important and rate-limiting enzyme for ATP degradation. Cancer therapies are in development to specifically target CD39, and being analyzed in early clinical trials (ClinicalTrials.gov: NCT04306900, NCT03884556). The evidence is growing to also consider CD73 as a potential target [73]. For example, Briceño et al. [74] recently published on the autocrine effect of CD73-mediated adenosine production, which limits the differentiation and metabolic fitness of CD8+ T cells. This finding has been supported by the fact that CD73-deficient cells, on the one hand, showed increased glucose uptake and higher mitochondrial respiration and, on the other hand, achieved a more effective reduction in tumor burden than wild-type cells after adoptive transfer into B16.OVA melanoma-bearing mice.

Nonetheless, adenosine metabolism is a complex and incompletely understood pathway. Apart from the above-mentioned better known enzymes, CD39 and CD73, it has now become clear that other enzymes may also play important roles in purinergic signaling, including members of the ectonucleotide pyrophosphatase/phosphodiesterase (ENPP) family, tissue nonspecific alkaline phosphatase (TNAP), and adenosine deaminase (ADA) [75]. This could be a potential pitfall for ongoing clinical trials, as the desired anti-tumor effect of CD39-CD73 axis blockade could lead to resistance to treatment by expanding shuffle signaling pathways.

The ectoenzymes CD39 and CD73 are found, not only on hematopoietic (mesenchymal stromal cells), cancer, and B cells [76], but also on tumor exosomes [77]. The latter appear to influence their own release in the sense of an autocrine effect via adenosine receptors [78]. Possibly, this leads to a high exosomal load. Our own analyses of exosomal burden in HNSCC, which have not yet been published, showed a significant correlation between the exosomal protein concentration and aggressiveness of the disease. Similarly, Pietrobono et al. [3] concluded that high extracellular levels of adenosine correlate with glioma aggressiveness. The underlying mechanisms suggest both direct interaction and modulation via detours through modulation of the mesenchymal stromal cell secretome [3]. However here, too, the relationships seem to be more complex and one looks in vain for a simple connection. Some previously published studies showed that millimolar concentrations of extracellular ADO significantly reduced the growth of pancreatic, hepatic, and colorectal cancers [79,80,81].

In general, the role of exosomes or EVs is still controversial, as some studies suggested that EVs can inhibit the immune system through adenosine and anti-inflammatory cytokine expression [43,82]. In contrast, there are also some reports of EVs with antitumor activity, and which have demonstrated immunogenic activity of EVs and the ability to induce T-cell-dependent antitumor immunity [83,84]. For example, in 2020 the research group led by Fabrício Figueiró demonstrated that glioma-derived extracellular vesicles expressing CD9, HSP70, CD39, and CD73, and producing adenosine, reduced glioma progression by modulating the tumor microenvironment. Thereby, a reduction in tumor size occurred by acting on cell proliferation [85].

Consequently, the relationship seems to be part of a complex network of interactions, in which the effect exerted by ADO on the players involved in the tumor micromileu depends on cell type, concentration, and type of receptor interaction [86]. In particular, the exact role of the individual adenosine receptors (A1, 2A, 2B) has not yet been conclusively determined.

## 7. Exosomes in Diagnostics: Highly Promising Candidates for Liquid Biopsy

Neuroradiology plays a central role in the diagnosis of gliomas. Furthermore, especially in the era of immunotherapies, imaging does not allow distinguishing pseudo-progression from pseudo-response with reasonable certainty. Recurrent biopsies are likewise not an option due to their invasiveness. With regard to circulating biomarkers, in addition to problems with sensitivity and specificity, there is also the problem with the blood–brain barrier (BBB) [87]. Although individual circulating tumor cells (CTCs) [88,89,90,91], and even quite recent clusters [92] of CTCs, have been found in peripheral blood from patients with GBM, it remains unclear to what extent CTCs are able to pass through the blood–brain barrier. Theoretically, CTCs can enable profiling of the whole-tumor genome, but they can also reflect only a single cell type of the heterogeneous tumor composition, whereas exosomes can reflect the complex heterogeneity of the whole tumor, as well as its adaptations to therapy [93,94]. 

The function of the BBB is to protect the central nervous system from toxins or infectious pathogens. Hypoxia, however, can damage this barrier and cause increased permeability [95], the genetic mechanism behind this is only partially understood. It is possible that exosomal VEGFR plays an active part in this process [96]. Studies by Zhao et al. [97] supported this assumption. They were able to show in an in vitro model that under hypoxic conditions glioblastomas release more VEGF-A carrying exosomes. These in turn had the ability to increase the permeability of the BBB by suppression of claudin-5 and other occluding proteins [97]. 

Banks et al. [98] examined ten different types of exosomes derived from very different cell lines (mouse, human, cancerous, non-cancerous) with respect to their ability to cross the blood–brain barrier. It was found that all the tested exosomes crossed the BBB without problems. However, there was a high variance concerning rates and vesicular-mediated mechanisms [98]. 

Consequently, it is a complex process with variable dynamics and it can be assumed that permeability of the BBB in GBM varies both in an interindividual and in a stage-dependent manner. Due to their small size, exosomes can also overcome an intact BBB [99,100] and are thus suitable for early diagnosis and genotyping, irrespective of the stage.

Several authors have already succeeded in detecting exosomes of glioblastomas peripherally [96,101,102,103]. Johan Skog et al. [96] already succeeded in 2008 in detecting the EGFRvIII status of glioblastomas using microvesicles drawn from peripheral blood. Even at this early stage in the field of liquid biopsy, the authors concluded that longitudinal blood sampling offered a way to monitor tumor genetic dynamics [96]. Fortunately, they were not alone in their success in the years that followed, other research groups also succeeded in detecting microvesicles such as GDEs in peripheral blood [71,72,73]. For instance, in 2018, Manda et al. presented exosomes as biomarkers for detecting EGFR-positive high-grade gliomas [103]. Already, the exosomal protein amount correlates with the aggressiveness of the glioma, which shows the potential of this measurement in diagnostics [104]. This could also be shown for other tumors. Ludwig et al. [105] observed that the protein quantities of extracellular vesicles (EVs) isolated from HNSCC patient plasma were significantly higher than in healthy donors. In addition, they showed that an increase in total plasma EVs correlates with disease activity [105]. For a more precise tumor diagnosis, however, much more information is needed. Interestingly, 90% of all patients with GBM showed aberrant expression of at least one of the following markers on the exosomal level: EGFR, EGRRvIII, podoplanin, and IDH1 [106]. In the field of exosomal diagnostics, proteins are the most frequently investigated exosomal cargo to date [107]. Nevertheless, other components, like mRNAs and miRNAs, also have great potential to become part of a diagnostic panel in the future.

There are also promising data in the field of exosomal microRNAs (miRNA) with regard to diagnosis and characterization of glioblastomas. MiRNAs play an important role as cellular regulators in a variety of physiological and pathological conditions. According to recent findings, miRNAs can be exchanged between cells via exosomes and their detection allows many conclusions to be drawn about the parental cell. In a widely published pilot study by Ebrahimkhani et al. [108] in 2018, exosomal microRNAs were isolated from the serum of glioblastoma patients and analyzed by unbiased deep sequencing. Exosomal RNA was superior to free RNA in terms of diagnostic predictability [108]. Similarly, a study by Daasi et al. demonstrated that the exosomal PD-L1 status, but not the soluble form of PD-L1, was of prognostic value for disease progression in HNSCC [109]. Furthermore, exosomes of hypoxic glioblastoma cells were found to contain miR-301a, a driver of resistance to radiotherapy. Tumor cells in normoxic conditions take up these exosomes and enter a state of radiotherapy resistance by influencing the Wnt/ß-catenin signaling pathway, analogous to plasmids in bacteria that transfer antibiotic resistance. 

More promising data came from Manterola et al. [110]. They found that the expression levels of one small noncoding RNA (RNU6-1) and two microRNAs (miR-320 and miR-574-3p) were significantly associated with GBM diagnosis. Here, RNU6-1 was consistently an independent predictor of GBM diagnosis [110]. A similarly promising indicator of glioma diagnosis and prognosis is exosomal miR-21, since its levels were shown to correlate notably with tumor recurrence or metastasis [111]. 

All these are increasing evidence for the high potential of small non-coding RNA signatures in microvesicles, which can be isolated from the serum of GBM patients. Exosomes are thus increasingly emerging as reliable differential diagnostic biomarkers in cancer, which could be a valuable addition to the current diagnostics, relying mainly on imaging. A long-term vision would be to apply these findings, not only for diagnostics, but also in a follow-up and therapeutic context [112].

However, until exosomes can be used analogously to, for example, PSA in prostate carcinoma, some more investigations will be necessary, because their role in tumors seems to be complex and anything but one-dimensional. For example, their molecular cargo can exert both pro- and antitumor effects [113,114]. Probably, some kind of panel of different partial analyses of exosomal cargo (DNA, mRNA, miRNA, proteins) could increase the accuracy, and thus become a diagnostic building block. 

Before clinical use can be considered here, however, a simple, standardized, and reproducible isolation method is needed.

## 8. Analysis of Exosomes for Diagnostic Purposes: Standardization as the Crucial Foundation

The increasingly deep understanding of exosomes and their potential application value, calls for reproducible isolation and enrichment. To date, most current isolation technologies cannot fully separate exosomes from lipoproteins with similar biophysical properties and other non-endosomal EVs [115]. In addition to the purity issue, maintaining biological integrity is also critical. Thus, we are currently faced with the difficulty of how best to enrich exosomes from biofluids and prepare them for downstream analyses.

The most commonly chosen isolation methods include ultracentrifugation and density gradient centrifugation, polymer precipitation, size-based isolation techniques, and immunoaffinity capture techniques. 

Ultracentrifugation (UC) is currently considered the gold standard for exosome extraction and separation. The basis of this isolation method is the size and density differences of the components of the sample. It is also suitable for the separation of large-volume sample components with significant differences in sedimentation coefficient [116]. In addition, the exosomes do not need to be labeled, which can avoid cross-contamination [117]. However, problematic in terms of reproducibility are the centrifugation time, centrifugal force, rotor type, and the parameters affecting the yield and purity of the target exosomes [116,118]. Density gradient centrifugation is usually used in combination with ultracentrifugation to improve exosome purity. A sucrose density gradient is usually used, but this is poor at distinguishing exosomes from retroviruses, due to their biophysical properties [119]. In addition, this method provides a high purity of exosomes, but the high viscosity caused by the sucrose solution leads to a reduction in the sedimentation speed and thus a higher time. 

Another approach is size-exclusion chromatography (SEC). The separation principle is that the macromolecules cannot enter gel pores and are thus eluted along gaps between the porous gels with the mobile phase, while the small molecules remain in the gel pores and are finally eluted from the mobile phase. Various exosome purification columns based on the SEC principle are now commercially available. The application of SEC is fast, simple, and inexpensive. However, the exosome isolates may be contaminated with other particles of similar size, resulting in reduced purity [117].

Polymer precipitation is another method that was originally used to isolate viruses. Polyethylene glycol (PEG) is usually used as the medium, and the exosomes are harvested under centrifugation conditions by reducing the solubility of the exosomes. The method is also suitable for large sample volumes and has relatively short analysis times. Nevertheless, the purity and recovery rate are relatively low, which can lead to false positive results. Since the resulting polymer is difficult to remove, it makes subsequent experimental functional analysis rather difficult [120].

Immunoaffinity chromatography (IAC) is based on the specific binding of antibodies and ligands to separate desired substances from heterogeneous mixtures. In principle, the antigens used in this method should be proteins with high abundance on the surface of exosome membranes, such as ESCRT complex-related proteins or proteins of the four-transmembrane protein superfamily. In addition to general components on the surface of exosomes, specific structures typical of certain cell types can also be target proteins. The latter is used to isolate exosomes of a specific origin. IAC can be used for qualitative and quantitative determination of exosomes and has good specificity and sensitivity [121]. 

However, the storage conditions of exosomes obtained by immunoaffinity chromatography are relatively challenging, and the method is not suitable for large-scale separation of exosomes.

Given the strong increase in interest in this field and thus various isolation techniques, we kindly refer the reader for a more detailed description of further methods to reviews on isolation techniques [115,122].

For different purposes and applications, different methods have advantages and disadvantages. However, to compare samples from several patients interindividually and intraindividually over a longer period of time, a standardized method is required. Many common methods such as ultracentrifugation, density gradient centrifugation, or precipitation are not practical in the clinical routine for many reasons already mentioned (including high time consumption, need for larger quantities, contamination, loss of exosomes). 

**Figure 2:**
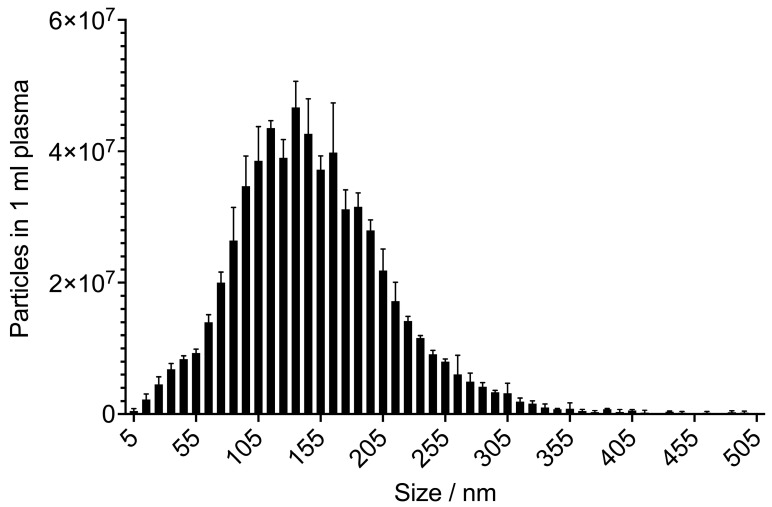
Biological triplicate of extracellular vesicles (EVs) from three cancer patients isolated with the EXÖBead® technique, showing the typical size range of exosomes between 30–150 nm. Average particle concentration with standard deviation in 1 mL plasma: 6.11 × 10^8^ ± 4.41 × 10^7^. Average particle size with standard deviation in 1 mL plasma: 138.30 ± 2.59. Analysis with a ZetaView® (Mebane, NC, USA): Nanoparticle Tracking Analyzer.

Thanks to the growing interest in this field, there is an increasing supply of commercial kits. After several years of experience with different techniques of exosome purification, our research group has succeeded in establishing a novel isolation method for obtaining exosomes from blood, and thus reducing the laboratory workload to a few hours. Here, we work with glycan particles based on galectin, which are able to capture exosomes in plasma and thus make ultracentrifugation unnecessary. The quality of exosomal isolation is monitored by electron microscopy and nano tracking analyses (ZetaViewer®, Mebane, NC, USA). The typical size range of exosomes is 30-150 nm (Figure 2). A functional assay for T-cell activation was able to show that the exosomes isolated in this way remain biologically active. 

Our results first showed that galectin-coupled magnetic beads, EXÖBead® (Biovesicle Inc., Taipei, Taiwan), can isolate EVs from plasma with low contamination of lipoprotein. In addition, we could also use a lactose-based elution buffer to successfully obtain the intact functional EVs after elution. A manuscript describing this method is under publication.

## 9. Exosomes in Therapy Monitoring: With Multiple Markers to Higher Sensitivity

Glioblastoma is a particularly aggressive tumor entity that has a highly variable response to therapy, which to date makes prediction regarding therapy response very difficult. The standardized diagnostics since the invention of cross-sectional imaging are CT, and especially MRI. The field of radiomics is making great efforts to provide more precise information and to better distinguish pseudo-progression from pseudo-regression. However, it is likely that the use of AI, while increasing prognostic accuracy, will always leave a residual uncertainty. As anywhere in life, it helps to look at things from a different angle to get closer to the matter at hand. There is evidence, for example, that exosomes adjust their content (including miRNA, mRNA, and proteins) during the course of the disease [123,124]. This allows monitoring changes during the therapy. 

Zeng et al. [125] investigated the effect of exosomal miR-151a expression level on temozolomide (TMZ) resistance. Using quantitative PCR analysis in two TMZ-resistant GBM cell lines, it was determined, that these cells secreted lower levels of miR-151a-containing exosomes. Conversely, overexpression of miR-151a sensitized chemoresistant GBM tumor cells to TMZ by suppressing the XRCC4 DNA repair pathway. Thus, obtaining miR-151 a-containing exosomes not only has diagnostic significance in terms of liquid biopsy to aid in the choice of therapy but could also be a component of treatment for TMZ-resistant GBM [125]. The critical role of XRCC4 in brain tumors had already been described by other research groups [126]. Yet, the exact function of XRCC4 in oncogenicity and TMZ resistance in GBM remains to be elucidated in more detail, before work can be done on clinical applications.

Radiotherapy is one of the established components of glioblastoma therapy, along with surgery and chemotherapy. Li et al. investigated whether the measurement of miRNAs can monitor the efficacy of radiotherapy in GBM patients [93]. For this purpose, exosomal miRNA levels were sequenced before and after radiotherapy in a cohort study. All miRNAs that showed significantly altered expression were examined. Different databases came into use to analyze the target genes of the corresponding miRNAs. 

A large proportion of the target genes were involved in the p53 signaling pathway and various known tumor progression pathways, suggesting that these miRNAs may play an impacting role in glioma development and progression via their effects on target genes. Thus, at the exosomal level, it has been possible to partially map the response of the tumor and its environment to radiotherapy, providing another step towards biomarkers for therapy monitoring.

In addition, especially in the monitoring of new therapies, the analyses of exosomes and their components can have an important role in therapy control. In our own study of patients enrolled in a glioma vaccination trial, we showed that the exosomal levels in serum correlated positively with the corresponding tumor stage. This translated into a decreasing exosome load in response to therapy, while higher levels were measured in clinical tumor increase. Exosomal immune-related protein levels also correlated positively with different grades of glioma. Furthermore, clinical response to the given tumor-related vaccine correlated with a change in serum exosomal immune-related gene expression, which gives them a promising role in therapy monitoring [49].

## 10. Exosomes in Therapy: Much Hope but Still a Long Way to Go

Fortunately, in recent years it has been found that exosomes, contrary to what has been assumed for decades, are not mere garbage cans of the cell. Since the first observations of these EVs in reticulocytes [127], there has been a tremendous increase in the understanding of exosomes as transmitters of cellular information. The fact that they can divulge much about their parental cells has led to intensive research into the diagnostic use of exosomes. Therapeutic use of exosomes is also anticipated to be very promising in the treatment of cancer, although research in this area is much less advanced.

Exosomes are a well-studied class of EVs known to transport proteins and nucleic acids and to protect their contents from proteases and RNAases through their double membrane. They are histocompatible, not recognized by the complement system, do not trigger adverse immune responses due to their self-derived origin, and their nanoscale size reduces their clearance from the mononuclear phagocyte system [128]. Biological therapeutics, including short interfering RNA and recombinant proteins, are susceptible to degradation, have limited ability to cross biological membranes, and can trigger adverse immune responses. For this reason, delivery systems for such drugs are under intense investigation. Exosomes possess a number of essential characteristics that make them extremely valuable as drug delivery vectors. Their advantageous structure allows them to deliver different types of molecules and target particular cells, due to transport specifity [129].

Especially in the context of brain tumors, the ability of exosomes to cross the blood–brain barrier plays a central role [99,100]. Using a transgenic zebrafish as a brain cancer model, Yang et al. [130] showed that exosomes also delivered anticancer drugs across the BBB and into the brain.

However, the complexity of exosomes also comes with certain risks, especially the potential for off-target effects, and is a major challenge on the way to translation to the clinic. In the following, the potential therapeutic applications will be briefly elaborated.

### 10.1. Exosome-Based Gene and Drug Delivery

Various techniques are used to load exosomes with the desired cargo. A distinction is made between exogenous loading and endogenous loading. In the latter case, the modifications take place during the formation of the exosomes. In the former, the exosomes are isolated and then modified by freeze-thaw cycles, incubation, sonication, extrusion, or electroporation.

The successful use of exosomes for the targeted delivery of siRNA has already been demonstrated by Alvarez- Erviti [131] et al., in 2011. The exosomes were harvested, purified, and loaded with siRNA against an important protein in Alzheimer pathogenesis (BACE1) by electroporation and systemically injected. This resulted in a decrease (55%) of the harmful β-amyloid 1–42 protein in the brain. This biotechnological approach to create exosome-based delivery systems was the first demonstration of an exosome-based drug delivery system that showed efficient in vivo delivery of siRNA [131]. Other promising candidates for GBM therapy are miRNAs. However, the challenges include identifying the most effective miRNAs and the delivery method. Hamideh et al. used a rat model of glioblastoma to administer exosomes loaded with a miR-21-sponge, which led to a significant reduction in the volume of the tumors [132]. Munoz et al. [133] found that anti-miR-9-releasing exosomes increased multidrug transporter expression and sensitivity to TMZ in drug-resistant GBM cells. Increased caspase activity and cell death rate in tumor cells was the hoped-for consequence [133]. 

Other ways to overcome chemotherapy resistance involve packaging the substance itself into exosomes. Exosomes loaded with paclitaxel led to an increase in cytotoxicity of almost 50-fold in multidrug-resistant neoplasms compared with paclitaxel without exosomes [134]. Doxorubicin has also been successfully packaged into exosomes and proposed as a potential module for tumor therapy [135]. 

However, as much as we know about exosomes, we are still far from overlooking the complexity of this group of MVs and cannot predict with sufficient certainty the effect of modifications on their behavior. It is also possible that not all components of exosomes will be required for their function, so an alternative strategy is to synthetically recreate these vesicles. Much work is already being done on this as well. As early as 2012, Kooijmans et al. [128] considered how functional exosome mimetics could be produced by assembling liposomes containing only the crucial components of natural exosomes. By using components that are already well characterized, the pharmaceutical acceptability of such systems can be greatly increased. However, it needs to be determined which exosomal components are suitable [128].

### 10.2. Exosomes as Therapeutic Agents

Natural killer (NK) cells are an important part of the first-line defense in controlling tumor growth and metastasis. Like all cells, NK cells emit biologically active EVs that reflect their protein and genetic repertoire, and allow assessing the current NK cell status in cancer patients. In turn, these NK-derived exosomes could contribute to the improvement of cancer therapy by interacting with tumor and/or immune cells. However, a better understanding of the exact interactions is still needed [136]. Other approaches shed light on exosomes from umbilical cord blood-derived human mesenchymal stem cells, which also have partial antitumor effects via regulation of MiR-10a-5p/PTEN signaling [137]. Jia et al. [138] showed that modification of exosomes can simultaneously produce good results for targeted imaging and therapy. Thereto, they loaded exosomes with curcumin and superparamagnetic iron oxide nanoparticles in the first step and conjugated the exosome membrane with neuropilin-1-targeted peptide (RGERPPR, RGE) by click chemistry in the second step. The engineered exosomes easily passed the BBB and were found to be suitable for simultaneous diagnosis and therapy in the orthopic glioma model and in glioma cells [138]. However, these approaches are still in their infancy and time will tell what will finally prevail.

### 10.3. Exosome-Based Immunotherapy and Exosome Blocking

Exosomes have great potential in the field of cancer immunotherapy, with the potential to become the most effective cancer vaccines, as well as targeted antigen/drug carriers. Due to their ability to induce tumor-specific immunity, they are being traded as potential cancer vaccines, with studies in animals and in the clinic [85]. Due to the dual properties of exosomes (they can both inhibit and promote cancer development) a particularly good understanding of the underlying mechanisms is needed. At this point, the reader is referred to the expanding literature on this topic (for example, Xu et al., 2020 [139], Shi et al., 2020 [140], and Sinha et al., 2021 [141]).

Attributing to TEX a tendency toward protumorgenic activity, despite their dual properties, a finding by Massachusetts colleagues Atai, Balaj, Skog, Breakfield, and Maguire is also worth noting. They showed that the incubation of glioma-cell-derived exosomes with heparin resulted in a reduced exchange between donating and receiving cells [142,143]. By all appearances, the blockade occurred at the surface. However, we are not aware of any publications on therapeutic approaches in this field.

## 11. Conclusions

In the early days of cancer research, the focus was mainly on the malignant cells themselves, but in the last decades the spectrum has broadened significantly, and much attention is paid to the tumor microenvironment with its immune cells and intercellular communication. EVs, especially exosomes, are part of a novel communication system, as biologically active, selectively produced particles [144]. Moreover, exosomes carry much of the information of their parental cells and are detectable in almost all body fluids. This makes them ideal candidates for liquid biopsies in cancer diagnosis, and therapy monitoring [145], which has led to exosome research becoming a new area of interest for researchers worldwide.

Recent studies have highlighted the role of exosomes in tumor-induced immunosuppression, and analysis of these pathways helps in a deeper understanding of how glioblastomas and other tumors interact with different classes of immune cells [9,27]. Particularly with respect to B cells, very little is known, and certainly more research is needed to better understand their role in the TME. In addition, the use of exosomes as diagnostic and prognostic markers in brain tumors has evolved significantly in recent years [140]. Compared to other liquid biopsy techniques, such as ctDNA or CTCs, exosomes are more copious in blood and show increased stability. Both are criteria that may be relevant in the busy work environment [146].

Nevertheless, there are still many challenges in the clinical application of exosomes. Their isolation and purification are not standardized, yet this is exactly what is needed when seeking to use exosomes on a large scale in clinical trials. In addition, more reliable biomarkers should be confirmed. In the analysis of single biomarkers, only a few have been suitable for application to date. In all likelihood, the future will be a diagnostic panel that combines multiple biomarkers.

There have also been promising results in therapeutic applications of exosomes in vitro and in animal studies, but several challenges still need to be overcome, such as ensuring biosafety and targeting efficacy, as well as avoiding adverse effects, before exosomes can be successfully implemented in cancer therapy.

However, we are confident that with advances in technology, we will see the clinical use of exosomes in the diagnosis, treatment, and prognosis of gliomas and other tumors.

## Data Availability

Data available in a publicly accessible repository.
